# Supplementary material for: Actomyosin forces and the energetics of red blood cell invasion by the malaria parasite Plasmodium falciparum
Source: PLoS Pathog. 2020 Oct 26;16(10):e1009007. doi: 10.1371/journal.ppat.1009007 (PMC7644091; doi:10.1371/journal.ppat.1009007)
Supplement: S1 Fig — A Exchanging the constitutive BIP promoter for the Pfmyoa promoter produced p230p-prMA-sfGFP parasites. Genotyping PCR showed a complete loss of the WT p230p locus (green half arrow) and gain of the integrated locus (IN, grey half arrow). B Live fluorescence microscopy of p230p-prMA-sfGFP parasites showing DNA (DRAQ5, magenta) and cytosolic sfGFP expression in merozoites/ring and schizont. Scale bars 1 μm. C Western blot of p230p-prMA-sfGFP parasites confirming sfGFP expression, although at a lower level than constitutive p230p-BIP-sfGFP. D Western blot of p230p-prMA-sfGFP parasite lysate, normalised by total protein level, at different stages of the asexual life cycle. Strong expression of sfGFP was present in late schizonts and early rings only, as expected for the Pfmyoa promoter, while control protein P. falciparum aldolase (PfALD) levels dip slightly in trophozoite stages. (PDF) [file ppat.1009007.s001.pdf]

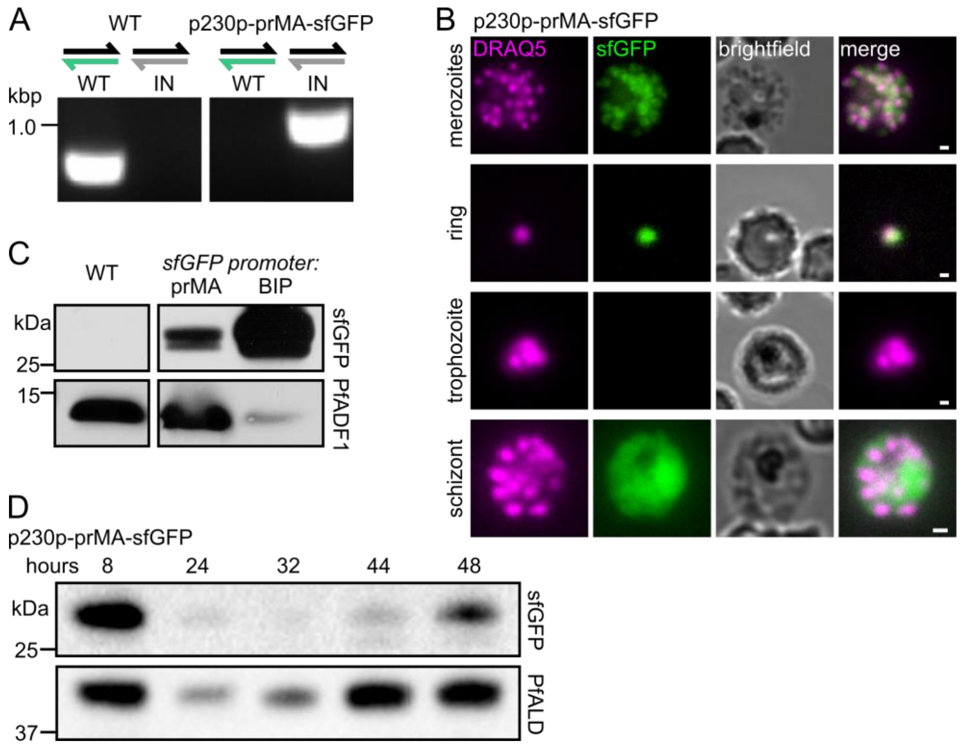

S1 Fig. Validation of *Pfmyoa* promoter system at the *p230p* locus

A Exchanging the constitutive *BIP* promoter for the *Pfmyoa* promoter produced p230p-prMA-sfGFP parasites. Genotyping PCR showed a complete loss of the WT *p230p* locus (green half arrow) and gain of the integrated locus (IN, grey half arrow). **B** Live fluorescence microscopy of p230p-prMA-sfGFP parasites showing DNA (DRAQ5, magenta) and cytosolic sfGFP expression in merozoites/ring and schizont. Scale bars 1 µm. **C** Western blot of p230p-prMA-sfGFP parasites confirming sfGFP expression, although at a lower level than constitutive p230p-BIP-sfGFP. **D** Western blot of p230p-prMA-sfGFP parasite lysate, normalised by total protein level, at different stages of the asexual life cycle. Strong expression of sfGFP was present in late schizonts and early rings only, as expected for the *Pfmyoa* promoter, while control protein *P. falciparum* aldolase (PfALD) levels dip slightly in trophozoite stages.
