# Supplementary material for: Actomyosin forces and the energetics of red blood cell invasion by the malaria parasite Plasmodium falciparum
Source: PLoS Pathog. 2020 Oct 26;16(10):e1009007. doi: 10.1371/journal.ppat.1009007 (PMC7644091; doi:10.1371/journal.ppat.1009007)
Supplement: S2 Fig — A To assess the consistency of the two biological repeats, each two technical repeats, performed for each line, since the statistical tests assume that all events are independent, PfMyoA-comp + DMSO was chosen as an example where both repeats (Exp4 and Exp6) had similar numbers of events. The proportion of successful invasion events in both experiments was the same (p>0.999, Fisher’s exact test, comparing pooled failures to Type A). B Comparing the deformation scores across the two experiments showed some differences, but not significant (p = 0.162, chi square test). C Comparing the distributions of each phase duration across the two experiments showed only small differences. The duration of internalisation was significantly higher in Exp6, but the difference was small (11.7 s to 12.7 s). Bars show median and interquartile range. Significance assessed for each phase by Mann-Whitney test, shown when p<0.5. (PDF) [file ppat.1009007.s002.pdf]

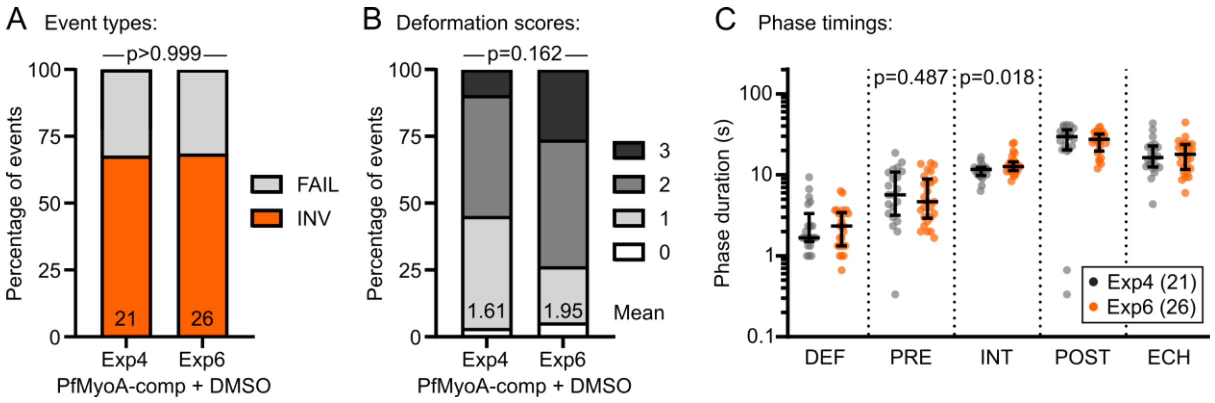

S2 Fig. Video microscopy of PfMyoA-comp, broken down by biological repeat to assess consistency

A To assess the consistency of the two biological repeats, each two technical repeats, performed for each line, since the statistical tests assume that all events are independent, PfMyoA-comp + DMSO was chosen as an example where both repeats (Exp4 and Exp6) had similar numbers of events. The proportion of successful invasion events in both experiments was the same (p>0.999, Fisher's exact test, comparing pooled failures to Type A). **B** Comparing the deformation scores across the two experiments showed some differences, but not significant (p=0.162, chi square test). **C** Comparing the distributions of each phase duration across the two experiments showed only small differences. The duration of internalisation was significantly higher in Exp6, but the difference was small (11.7 s to 12.7 s). Bars show median and interquartile range. Significance assessed for each phase by Mann-Whitney test, shown when p<0.5.
