# Supplementary material for: Actomyosin forces and the energetics of red blood cell invasion by the malaria parasite Plasmodium falciparum
Source: PLoS Pathog. 2020 Oct 26;16(10):e1009007. doi: 10.1371/journal.ppat.1009007 (PMC7644091; doi:10.1371/journal.ppat.1009007)
Supplement: S3 Fig — A From all lines after DMSO treatment, comparing Type A events to Type B events shows a significantly shorter deformation period and slower internalisation, with a trend to a longer pause before internalisation, consistent with either a merozoite producing less force or an RBC providing more resistance to invasion. Bars show median and interquartile range. Significance assessed for each phase by Mann-Whitney test, shown when p<0.5. B Comparing Type B failures from all DMSO- or RAP-treated lines shows no difference in the delay from start of internalisation to merozoite ejection. Bars show median and interquartile range. Significance assessed by Mann-Whitney test. C Example images of Type B failure where the invasion pore remained visible, hence not resealed (A = attachment, E = ejection); the incompletely-internalised merozoite continuing to move in a swirling pattern within the invagination; an ejection from PfMyoA-K764E after RAP treatment, where Type B failure was more common; and an event where resealing after internalisation was prevented by tethering to a sister merozoite. (PDF) [file ppat.1009007.s003.pdf]

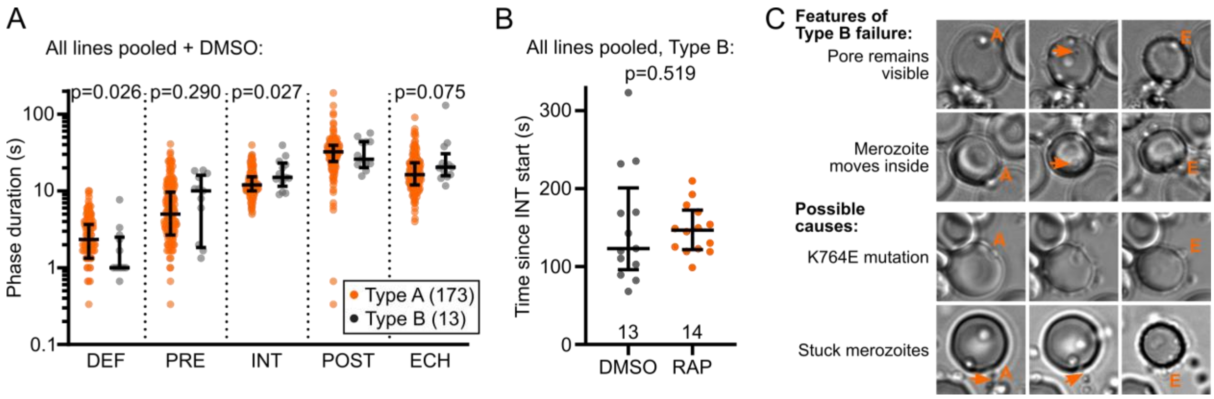

S3 Fig. Type B events show slower internalisation

A From all lines after DMSO treatment, comparing Type A events to Type B events shows a significantly shorter deformation period and slower internalisation, with a trend to a longer pause before internalisation, consistent with either a merozoite producing less force or an RBC providing more resistance to invasion. Bars show median and interquartile range. Significance assessed for each phase by Mann-Whitney test, shown when p<0.5. **B** Comparing Type B failures from all DMSO- or RAP-treated lines shows no difference in the delay from start of internalisation to merozoite ejection. Bars show median and interquartile range. Significance assessed by Mann-Whitney test. **C** Example images of Type B failure where the invasion pore remained visible, hence not resealed (A= attachment, E= ejection); the incompletely-internalised merozoite continuing to move in a swirling pattern within the invagination; an ejection from PfMyoA-K764E after RAP treatment, where Type B failure was more common; and an event where resealing after internalisation was prevented by tethering to a sister merozoite.
