# Supplementary material for: Actomyosin forces and the energetics of red blood cell invasion by the malaria parasite Plasmodium falciparum
Source: PLoS Pathog. 2020 Oct 26;16(10):e1009007. doi: 10.1371/journal.ppat.1009007 (PMC7644091; doi:10.1371/journal.ppat.1009007)
Supplement: S1 Table — (PDF) [file ppat.1009007.s009.pdf]

S1 Table. Oligonucleotides used in this study for genotyping PCR and sequencing

#	Locus	Part	Sequence	Source
1	*p230p*	WT[a]/IN[b]-F	ACCATCAACATTATCGTCAG	[1]
2	*p230p*	WT-R	TCTTCATCAGCCTGGTAAC	[1]
3	*p230p*	IN-R	CATTTACACATAAATGTCACAC	[1]
4	PfMyoA-K764E	IN-F	GTTCATTGATTGGATCTCAGTTC	This study
5	PfMyoA-K764E	IN-R	GGATACGAGCCAGCATAGTC	This study
6	PfMyoA-cKO	IN/EX[c]-F	GGTCGTTTCATGCAGTTGGT	[2]
7	PfMyoA-cKO	IN-R	GCCAGCCACGATAGCCGCGCTGCCTCGTCCTGCAGT TCATTCAGGGCACCGGACAGGTCG	[2]
8	PfMyoA-cKO	EX-R	ACCTTCACCCTCTCCACTGAC	[2]
9	PfMyoB-cKO	WT/IN/EX-F	ATGGGATCGAAAAGGGTGGT	This study
10	PfMyoB-cKO	WT-R	TCCATGATCACTCGTCCTCAC	This study
11	PfMyoB-cKO	IN-R	AAGTCTCCACAATTGATAAAGAG	This study
12	PfMyoB-cKO	EX-R	TTATTTGTACAGTTCATCCATACC	This study
13	pDC2-p230p-BSD	Seq[d] (*bsd*)	TTTTTGTAATTTCTGTGTTTATG	This study
14	pDC2-generic	Seq (gRNA)	AAGCACCGACTCGGTGCCAC	Marcus Lee
15	p230p-prMA-sfGFP	Seq (*sfgfp*)	TGAACCATACGGGTTGTTG	This study
16	p230p-prMA-MyoA	Seq (*myoa*)	CTCCTGGAGCCAAGCAC	This study

[a]WT = wild type locus, [b]IN = integrated locus, [c]EX = excised locus, [d]Seq = sequencing

References

1. Ashdown GW, Dimon M, Fan M, Sánchez-Román Terán F, Witmer K, Gaboriau DCA, et al. A machine learning approach to define antimalarial drug action from heterogeneous cell-based screens. Sci Adv. 2020;6: eaba9338. doi:10.1126/sciadv.aba9338
2. Robert-Paganin J, Robblee JP, Auguin D, Blake TCA, Bookwalter CS, Krementsova EB, et al. Plasmodium myosin A drives parasite invasion by an atypical force generating mechanism. Nat Commun. 2019;10: 3286. doi:10.1038/s41467-019-11120-0
