# Supplementary material for: Actomyosin forces and the energetics of red blood cell invasion by the malaria parasite Plasmodium falciparum
Source: PLoS Pathog. 2020 Oct 26;16(10):e1009007. doi: 10.1371/journal.ppat.1009007 (PMC7644091; doi:10.1371/journal.ppat.1009007)
Supplement: S2 Table — (PDF) [file ppat.1009007.s010.pdf]

S2 Table. Oligonucleotides used in this study for cloning PCR and guide RNAs

#	Construct	Part	Sequence	Source
17	pDC2-p230p-BSD	BSD-F	ATATCCATGGCCAAGCCTTTGTCTCAAGAAGAATCC	This study
18	pDC2-p230p-BSD	BSD-R	ATATCCGCGGTTAGCCCTCCCACACATAAC	This study
19	p230p-prMA-sfGFP	prMA-F	ATATCCGCGGAGCATGCACAATTAAGAAGACG	This study
20	p230p-prMA-sfGFP	prMA-R	ATATGCTAGCTTTTTTTTTTTTTTTATAAATATGAAAAG	This study
21	p230p-prMA-PfMyoA	MyoA-F	CTTTTCATATTTATAAAAAAAAAAAAAAAGCTAGCATGGCCGTTACCAACG	This study
22	p230p-prMA-PfMyoA	MyoA-R	GATATTTACTTATTTATTTTATTCTGCATATTTAAAAATCCTGCAGTTAGACCGCATAATCCGG	This study
23	p230p-prMA-K764E	SDM[a]-F	GCAAGAGGGTGCTGAAATTTTAACAAAAATAC	This study
24	p230p-prMA-K764E	SDM-R	ATTTTTGTTAAAATTTCAGCACCCTCTTGC	This study
25	p230p-prMA-S19A	SDM-F	GTGAGGAGAGTAGCTAACGTGGAGG	This study
26	p230p-prMA-S19A	SDM-R	CAAAAGCCTCCACGTTAGCTACTCTCCTC	This study
27	p230p-prMA-DN	MyoA-F	CTTTTCATATTTATAAAAAAAAAAAAAAAGCTAGCATGAACGTGGAGGCTTTTGATAAATC	This study
28	pUC-PfMyoB-CK	HR[b]-F	CAGTCACGACGTTGTAAAACGACGGCCAGTGAATTCTTAGAGAATTTCATAGGTATTTTGG	This study
29	pUC-PfMyoB-CK	HR-R	TGCGTAATCCGGTACATCATATGGGTACATTTCATGCTCTTTTATATATTTGTACTTAC	This study
30	pUC-PfMyoB-CK	SF[c]-F	ATGTACCCATATGATGTACCG	This study
31	pUC-PfMyoB-CK	SF-R	TTATTTGTACAGTTCATCCATACC	This study
32	pUC-PfMyoB-CK	3UTR[d]-F	ACGCATGGTATGGATGAACTGTACAAATAACTCGAGAAATCGGGAAAATAAAATGG	This study
33	pUC-PfMyoB-CK	3UTR-R	TATGACCATGATTACGCCAAGCTTGCATGCCTGCAGGTCGTCCCAATCATTTTTC	This study
34	pDC2-p230p-hDHFR	gRNA[e]-F	TATTAGGCTGATGAAGACATCGGG	[1]
35	pDC2-p230p-hDHFR	gRNA-R	AAACCCCGATGTCTTCATCAGCCT	[1]
36	pDC2-pfmyob-hDHFR-1	gRNA-F	TATTGTATAAGATAGGAAAAAGCA	This study
37	pDC2-pfmyob-hDHFR-1	gRNA-R	AAACTGCTTTTTCCTATCTTATAC	This study
38	pDC2-pfmyob-hDHFR-2	gRNA-F	TATTGTAGAAGCATTTGACAAAAG	This study
39	pDC2-pfmyob-hDHFR-2	gRNA-R	AAACCTTTTGTCAAATGCTTCTAC	This study

[a]SDM = site-directed mutagenesis, [b]HR = homology region, [c]SF = synthetic fragment, [d]3UTR = 3' un-translated region, [e]gRNA = guide RNA.

References

1. Ashdown GW, Dimon M, Fan M, Sánchez-Román Terán F, Witmer K, Gaboriau DCA, et al. A machine learning approach to define antimalarial drug action from heterogeneous cell-based screens. Sci Adv. 2020;6: eaba9338. doi:10.1126/sciadv.aba9338
